# The female reproductive tract microbiotas, inflammation, and gynecological conditions: mechanisms, therapeutic advances, and future perspectives

**DOI:** 10.3389/fmicb.2026.1863564

**Published:** 2026-06-16

**Authors:** Xingchen Liu, Jingjing Xu, Zixuan Fan, Jun Cai, Dandan Zuo, Fang Wang

**Affiliations:** 1 Department of Gynecology, Xinyang Central Hospital, Xinyang, Henan, China; 2 School of Medicine, Henan University, Kaifeng, China; 3 College of Traditional Chinese Medicine, Guangzhou University of Chinese Medicine, Guangzhou, Guangdong, China; 4 Department of Anesthesiology, Xinyang Central Hospital, Xinyang, Henan, China; 5 Department of Otolaryngology, Xinyang Central Hospital, Xinyang, Henan, China

**Keywords:** female reproductive tract microbiome, gynecological diseases, inflammatory response, *Lactobacillus*, vaginal dysbiosis

## Abstract

The female reproductive tract (FRT), particularly the vagina, has traditionally been regarded as a sterile environment. However, it is now recognized that the vagina is a complex, dynamic ecosystem dominated by *Lactobacillus* species, which maintain internal homeostasis through acid production, competitive exclusion, and immune regulation. Microbial dysbiosis, characterized by a loss of *Lactobacillus* dominance, increased microbial diversity and overgrowth of pathogenic bacteria, triggers chronic low-grade inflammation by disrupting physical barriers, activating pattern recognition receptors (PRRs) and secreting pro-inflammatory metabolites. This links to various gynecological conditions, including bacterial vaginosis (BV), pelvic inflammatory disease (PID), persistent human papillomavirus (HPV) infection and cervical cancer, endometriosis, infertility, and poor outcomes of assisted reproductive technologies, as well as adverse pregnancy outcomes such as preterm birth. The microbiota of the FRT is shaped by the menstrual cycle, hormones, behavior, antibiotics, stress, and genetic factors throughout the life course. The development of microbiota-based diagnostic biomarkers and therapeutic interventions to promote female reproductive health is supported by elucidating the mechanisms of this axis, which also helps clarify the pathophysiology of these diseases.

## 1 Introduction

The female reproductive tract (FRT), especially the vagina, has long been considered to be a sterile environment according to traditional views (Baker et al., 2018; Goldenberg et al., 2008; Hansen et al., 2014). In fact, it is a complex and dynamic ecosystem composed of bacteria, fungi, viruses, and their metabolites, in conjunction with host cells and immune components (Bradford and Ravel, 2017; Happel et al., 2020; Madere and Monaco, 2022). Among these, the vaginal microbiota is the largest and best-defined constituent of the FRT, typically characterized by the prevalence of *Lactobacillus* species (Chen et al., 2017; Gholiof et al., 2022). These symbiotic microorganisms are not mere “residents”; they play indispensable roles in maintaining local health through close interactions with the reproductive tract epithelial cells. They metabolize glycogen to generate lactic acid, which keeps the vagina acidic (pH 2.8–4.2) and inhibits the proliferation of potential pathogens (Alakomi et al., 2000; Greenbaum et al., 2019; O’Hanlon et al., 2011, 2013; Witkin and Linhares, 2017); they compete for adhesion sites and produce antimicrobial substances, such as bacteriocins, to directly resist pathogen invasion (Aroutcheva et al., 2001); and they continuously “communicate” with the host immune system, participating in the regulation of local immune responses and maintaining a moderate immune quiescent state, thereby ensuring reproductive tract homeostasis (Gholiof et al., 2022). However, this delicate balance is not static (Gholiof et al., 2022). Microbial dysbiosis is defined as the absence of *Lactobacillus* dominance, abnormal elevation in microbial diversity, and overgrowth of specific pathogenic genera (Chee et al., 2020). Increasing evidence indicates that dysbiosis of the reproductive tract microbiota is closely associated with local and systemic chronic low-grade inflammation (Gholiof et al., 2022). Dysbiotic microbiota can activate pattern recognition receptors, such as Toll-like receptors, in epithelial cells through their pathogen-associated molecular patterns, triggering signaling pathways such as nuclear factor kappa B (NF-κB), leading to excessive production of pro-inflammatory cytokines, such as interleukin-1β (IL-1β), interleukin-8 (IL-8), tumor necrosis factor-α (TNF-α), and chemokines (Anahtar et al., 2015; Villa et al., 2020). This inflammatory state not only damages the integrity of the reproductive tract epithelial barrier, increasing susceptibility to sexually transmitted pathogens, such as HIV, but also creates a persistent pro-inflammatory microenvironment (Dabee et al., 2021; Kenyon et al., 2013; van de Wijgert and Jespers, 2017). Based on this, we propose the “microbiota-immune-inflammation” axis as a key framework for understanding the occurrence and development of various gynecological diseases. Current research has clearly demonstrated that microbial dysbiosis and its associated inflammatory response constitute a “common soil” linking a range of gynecological diseases (Gholiof et al., 2022). From common bacterial vaginosis to endometriosis, infertility, especially embryo implantation failure in assisted reproduction, and even malignant tumors such as endometrial, ovarian, and cervical cancers, characteristic microbiota disorder and upregulation of inflammatory markers have been observed. For example, in patients with endometriosis, specific pathogenic bacteria are enriched in the reproductive tract microbiota, mutually promoting the peritoneal inflammatory microenvironment (Jiang et al., 2021); in infertile women, a reduced abundance of *Lactobacillus* bacteria and an increased presence of other species in the endometrium may impede embryo implantation by provoking local inflammatory responses (Kyono et al., 2018; Moreno et al., 2016); in gynecological cancers, chronic inflammation associated with dysbiosis may contribute to tumorigenesis through mechanisms such as inducing genomic instability and promoting cell proliferation and angiogenesis (Gholiof et al., 2022). Therefore, in-depth exploration of the specific mechanisms of the “microbiota-immune-inflammation” axis in different gynecological diseases not only helps to elucidate their pathophysiological basis but also provides a highly promising direction for developing novel diagnostic markers and intervention strategies based on microbiota modulation (Molina et al., 2020).

## 2 Female genital microbiota: composition, function, and homeostasis

### 2.1 Definition of health status: *Lactobacillus*-dominant communities

The female genital microbiota plays a central role in maintaining local homeostasis and defending against pathogen invasion (Gholiof et al., 2022). Among these, the vaginal microbiota–the largest in biomass, approximately 10^10^–10^11^ bacteria, and most extensively studied–is typically correlated with a “healthy” state, marked by reduced bacterial diversity and predominant colonization by *Lactobacillus* species (Chen et al., 2017; Gholiof et al., 2022). As Gram-positive, anaerobic rod-shaped bacteria, *Lactobacillus* species convert vaginal glycogen derivatives into lactic acid, maintaining a vaginal pH in the acidic range of 2.8–4.2 (Greenbaum et al., 2019; O’Hanlon et al., 2013; Witkin and Linhares, 2017). This low pH environment effectively inhibits the growth of various potential pathogens (Alakomi et al., 2000; O’Hanlon et al., 2011, 2013). Beyond acid production, *Lactobacillus* maintains host health through multiple mechanisms, including attaching to vaginal epithelial cells, forming a physical barrier through competitive occupation of spatial sites, synthesizing bacteriocins and other compounds toxic to other bacteria, and continuously interacting with the host immune system to assist in regulating inflammatory responses and maintaining immune quiescence (Aroutcheva et al., 2001; Gholiof et al., 2022). Research indicates that the vaginal microbiota of women of reproductive age could be classified into distinct community state types (CSTs), most of which are dominated by specific *Lactobacillus* species. *Lactobacillus crispatus* dominates CST-I, *Lactobacillus gasseri* dominates CST-II, *Lactobacillus iners* dominates CST-III, and *Lactobacillus jensenii* dominates CST-V (France et al., 2020; Ravel et al., 2011; Table 1). Notably, the protective effect conferred by *Lactobacillus iners* appears to be lower than that of other *Lactobacillus* species (Petrova et al., 2017; Table 1). However, this traditional definition of “health” is being challenged. Studies indicate that some asymptomatic and clinically healthy women exhibit diverse microbial compositions or anaerobically dominated vaginal microbiomes like CST-IV (Table 1), suggesting that individualized “healthy” baselines may vary and be influenced by multiple factors including ethnicity, geography, and sociodemographic background (Ravel et al., 2011; Zhou et al., 2007).

**TABLE 1:** Community state types (CSTs) of vaginal microbiota and their dominant *Lactobacillus* species.

CST	Dominant *Lactobacillus* species	Other features
CST-I	*Lactobacillus crispatus*	Typically associated with the most stable healthy state.
CST-II	*Lactobacillus gasseri*	Represents a healthy state.
CST-III	*Lactobacillus iners*	A healthy state, but its protective effect may be weaker than that of other *Lactobacillus* species.
CST-IV	Non-*Lactobacillus*-dominant	High diversity, lacking *Lactobacillus* dominance; represents a state of microbial dysbiosis and is associated with inflammation.
CST-V	*Lactobacillus jensenii*	Represents a healthy state.

### 2.2 Microbiome dysbiosis: bacterial vaginosis and related conditions

Microbiome dysbiosis refers to a state where the balance of the reproductive tract’s microbial community is disrupted. The most common manifestation of dysbiosis in the vaginal environment is the reduction of *Lactobacillus* dominance and an abnormal increase in microbial diversity (Chee et al., 2020). Bacterial vaginosis (BV), mainly characterized by reduced *Lactobacilli* and overgrowth of anaerobic and facultative anaerobic bacteria, is the only clinical diagnosis directly associated with the vaginal microbiome and is the most common vaginal disorder in women of reproductive age, including *Gardnerella vaginalis*, *Prevotella* spp., *Mobiluncus* spp., *Atopobium* spp., and *Sneathia sanguinegens* (Gajer et al., 2012; Marrazzo et al., 2002). BV correlates closely with elevated vaginal pH (>4.5), the presence of clue cells, and increased levels of inflammatory markers (Aldunate et al., 2015; Gholiof et al., 2022). Dysregulated vaginal microbiota form biofilms, which the immune system and antibiotics can’t fully clear, leading to prolonged infections and high BV recurrence rates (Bradshaw et al., 2006; Cerca et al., 2005). In addition to typical BV, CST-IV also represents a state of vaginal dysbiosis, inherently indicating a lack of *Lactobacillus* dominance (Gajer et al., 2012). Studies indicate that inflammatory features of the vaginal microbiota correlate strongly with increased microbial diversity, and CST-IV is a stronger predictor of inflammation than BV infections (Dabee et al., 2021; Lennard et al., 2017).

### 2.3 Dynamic nature and influencing factors

The female reproductive tract microbiome is not static but undergoes dynamic regulation by multiple intrinsic and extrinsic factors, including the menstrual cycle, hormones, behaviors, and environmental influences. Longitudinal studies indicate that while the vaginal microbiome remains relatively stable overall, its composition undergoes transient alterations in response to physiological states (Gajer et al., 2012; Ravel et al., 2013; Santiago et al., 2011; Srinivasan et al., 2010). Menstrual blood changes the pH and substrates of the vagina, thereby influencing the composition of its microbes (Eschenbach et al., 2000; Moosa et al., 2020). During pregnancy, hormonal levels tend to rise, stabilizing the vaginal microbiota and favoring a *Lactobacillus*-dominant state (DiGiulio et al., 2015; Romero et al., 2014). Major hormonal shifts across life stages profoundly influence the microbiota: Prepuberty features a predominantly anaerobic vaginal microbiota; transition to *Lactobacillus* dominance upon entering reproductive age; post-menopause, declining estrogen levels cause the microbiota to revert to an anaerobic-dominant composition (Alvarez-Olmos et al., 2004; Brotman et al., 2018; Shen et al., 2016). Behaviors such as sexual intercourse and the use of vaginal douches are linked to a reduction in vaginal lactobacilli (Gholiof et al., 2022; Moosa et al., 2020). Antibiotics disrupt endogenous Lactobacilli, potentially causing dysbiosis (Bradshaw and Sobel, 2016; Verwijs et al., 2020). Research indicates elevated perceived stress levels correlate with increased BV risk (Turpin et al., 2021). Host genetic factors, such as Toll-like receptor 4 (TLR4) gene polymorphisms, may also influence vaginal microbiota composition (Genc et al., 2004).

## 3 Core mechanisms of inflammation induced by microbial dysbiosis

Dysbiosis of the FRT microbiota disrupts local homeostasis and triggers inflammation through a series of interconnected physical, immune, and metabolic mechanisms. These mechanisms collectively form the pathophysiological basis linking microbial imbalance to various gynecological diseases.

### 3.1 Physical barrier disruption

**FIGURE 1:**
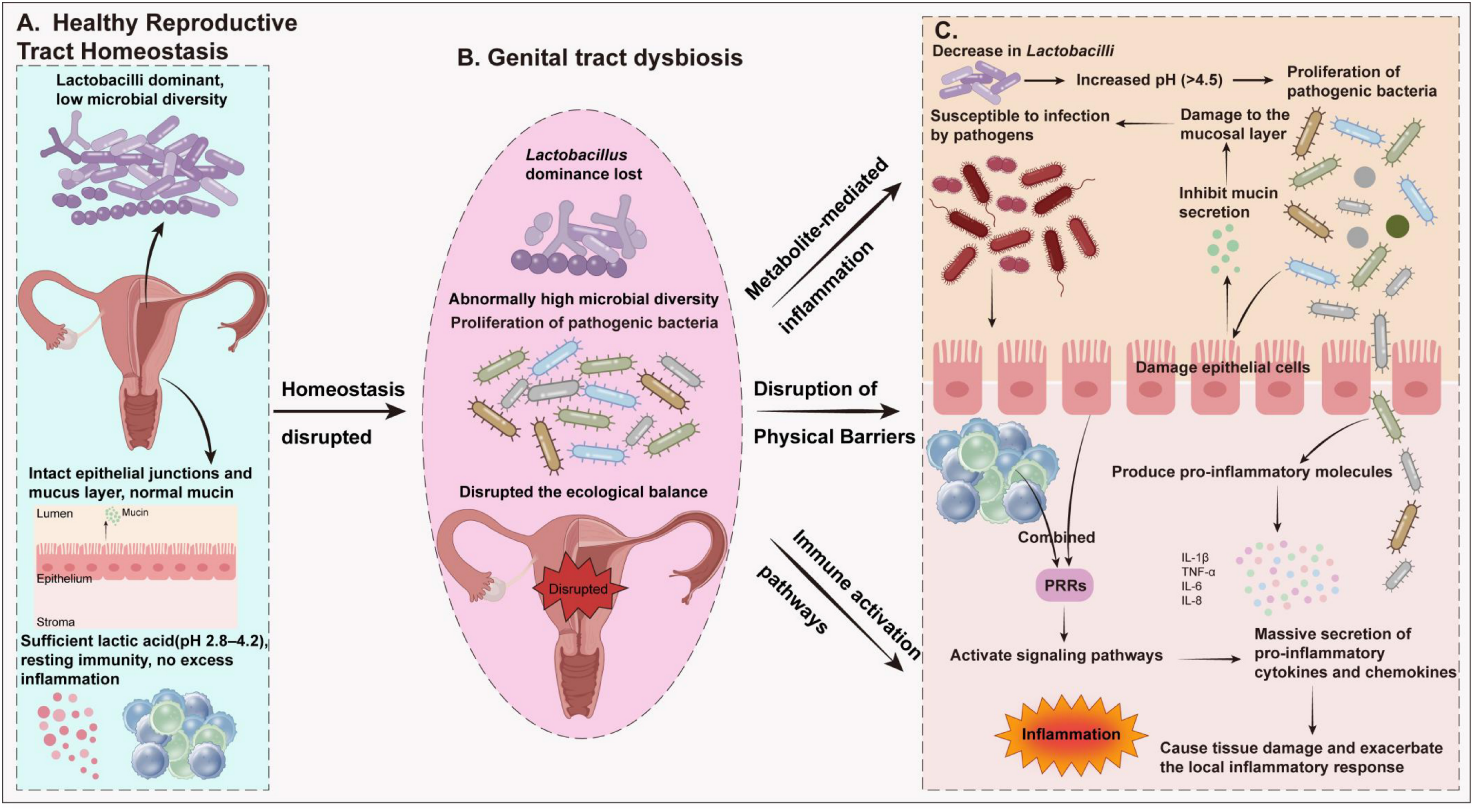
Core mechanisms linking reproductive tract dysbiosis to inflammation. **(A)** Healthy homeostasis with dominant *Lactobacillus*, intact epithelium, low diversity. **(B)** Dysbiosis with loss of *Lactobacillus* and increased pathogenic diversity. **(C)** Consequences: mucosal damage, immune activation, and pro-inflammatory cytokine release.

The healthy FRT epithelial cells and their surface mucus layer form the first line of defense against pathogens. Symbiotic microbiota, particularly lactobacilli, help maintain tight and adherens junctions between epithelial cells by producing mucins and other substances (Baker et al., 2018; Radtke et al., 2012; Figure 1A). However, microbial dysbiosis can directly damage this physical barrier. Studies have shown that an increased variety of vaginal microbes is associated with an impaired reproductive tract epithelial barrier, high levels of mucosal inflammation, and an elevated risk of contracting sexually transmitted infections, including HIV (Kenyon et al., 2013; van de Wijgert and Jespers, 2017; Figures 1B, C). The loss of barrier integrity thus creates conditions for pathogen invasion and persistent local inflammation.

### 3.2 Immune activation pathways

When physical barriers are breached, dysregulated microbiota and their products can directly activate the host’s immune recognition system, triggering a cascade of inflammatory responses. Epithelial cells and immune cells lining the FRT express various pattern recognition receptors (PRRs), such as Toll-like receptors (TLRs) and NOD-like receptors (Carvalho et al., 2012; Horne et al., 2008; Mitchell and Marrazzo, 2014; Usluogullari et al., 2014; Witkin et al., 2007; Figure 1C). When microbes trigger these receptors through their pathogen-associated molecular patterns (PAMPs), such as lipopolysaccharides and lipoproteins, it instigates the release of pro-inflammatory cytokines and chemokines (Gholiof et al., 2022). For example, TLR4 is expressed in the cervix, endometrium, and fallopian tubes and may help regulate immune tolerance in the FRT (Fazeli et al., 2005). Anahtar et al. (2015) posited that cervical-vaginal bacteria could stimulate inflammation by being recognized by epithelial and antigen-presenting cells through the NF-κB and Toll-like receptor pathways. The activation of PRRs ultimately leads to the secretion of multiple potent pro-inflammatory cytokines. Studies show that when PRRs on FRT epithelial cells are activated by microbes, they secrete cytokines including IL-1β, TNF-α, IL-6, and IL-8 (Villa et al., 2020; Figure 1C). Among these, IL-1β is a pivotal inflammatory mediator that not only directly promotes inflammation but also directs the initial CD4+ T cells toward a pro-inflammatory phenotype *in vitro* and prompts the expression of other pro-inflammatory cytokines (Hebel et al., 2011). Meanwhile, chemokines, such as IL-8, secreted by epithelial cells are responsible for the recruitment and activation of cells of both the innate and adaptive immune systems, including macrophages and CD8+ cytotoxic T cells (Villa et al., 2020). For example, the site of inflammation attracts activated CCR5+ CD4+ T cells, which are the target cells for HIV infection, via a process involving secreted chemokines (Gholiof et al., 2022).

### 3.3 Metabolite-mediated inflammation

Microbiome metabolic activities profoundly influence the local microenvironment, and their dysregulation directly participates in inflammation regulation. In a healthy state, dominant lactobacilli metabolize glycogen to produce lactic acid, maintaining the vaginal acidic environment (pH 2.8–4.2) and inhibiting the growth of harmful bacteria (Figure 1A). Dysbiosis leads to a reduction in lactobacilli, decreased lactic acid production, and an increase in vaginal pH (>4.5) (Figure 1C). It is noteworthy that vaginal short-chain fatty acids (SCFAs), such as butyrate and succinate, exhibit a pro-inflammatory effect and weaker antimicrobial activity, unlike the gut microbiota, which produces anti-inflammatory SCFAs (Amabebe and Anumba, 2020; Delgado-Diaz et al., 2020; Furusawa et al., 2013; Klynstra et al., 1967). During vaginal microbiota dysbiosis, elevated SCFA levels could facilitate IL-8 and TNF-α production induced by TLR2 and TLR7 ligands in a dose-dependent manner. Additionally, the pro-inflammatory effects of SCFAs are partly driven by their ability to stimulate the production of reactive oxygen species (ROS) (Gholiof et al., 2022; Mirmonsef et al., 2012). Moreover, BV-associated anaerobic bacteria could synthesize amines, such as cadaverine and putrescine, which not only cause typical symptoms but also further elevate pH levels, impair epithelial cell function, and potentially stimulate immune responses (Figure 1C).

## 4 Microbial dysbiosis, inflammation, and specific gynecological diseases

The balance between FRT microbiota and the immune system is crucial for maintaining reproductive health, and disruption of this balance and the accompanying inflammatory response are common features in the onset and progression of various gynecological diseases (Gholiof et al., 2022).

### 4.1 Bacterial vaginosis and pelvic inflammatory disease

Microbial dysbiosis first disrupts the epithelial physical barrier, activates PRR-mediated immune signaling, and alters pro-inflammatory metabolites, these three pathways collectively drive the pathogenesis of BV and its progression to PID. BV is a classic manifestation of vaginal microbiota dysbiosis, characterized by a loss of *Lactobacillus* dominance and overgrowth of anaerobic bacteria (Gholiof et al., 2022). BV is not only a localized condition but also serves as a “bridge” linking lower genital tract infections to upper genital tract inflammation and complications (Gholiof et al., 2022). Traditionally, the upper reproductive tract was regarded as sterile, but this view has been contested (Baker et al., 2018; Goldenberg et al., 2008). Studies have shown that bacteria can travel upward from the cervix (Hansen et al., 2014). Transplanting vaginal microbiota from patients with chronic endometritis into rats leads to exacerbated uterine inflammation, manifested by elevated levels of TNF-α and IL-1β (Gholiof et al., 2022). Similarly, when BV-associated bacteria, such as *Prevotella bivia*, are transplanted into the rat vagina, these bacteria can ascend to the uterus, thereby triggering inflammation and endometritis-like lesions (Wang et al., 2021), providing direct evidence that BV leads to pelvic inflammatory disease (PID). Furthermore, BV-associated microbial dysbiosis disrupts the stability of the reproductive tract environment (Campisciano et al., 2018; Łaniewski et al., 2020). In the presence of BV, vaginal epithelial cells are damaged and undergo increased apoptosis, leading to a disruption in the integrity of the epithelial barrier and thereby inducing chronic inflammation, manifested by elevated concentrations of pro-inflammatory cytokines, such as IL-1β and IL-8, in cervical and vaginal secretions (Aldunate et al., 2015; Gholiof et al., 2022; O’Hanlon et al., 2020; Roselletti et al., 2020). The compromised epithelial barrier and persistent inflammatory environment create conditions conducive to the invasion and ascension of other pathogens, such as *Chlamydia trachomatis* and *Neisseria gonorrhoeae*, thereby increasing the risk of PID, infertility, and adverse pregnancy outcomes (Dabee et al., 2021).

### 4.2 Persistent HPV infection, cervical cancer and endometrial cancer

The epithelial barrier damage, immune overactivation, and pro-inflammatory metabolite accumulation caused by microbiota dysbiosis create a chronic inflammatory microenvironment that directly promotes persistent HPV infection and cervical lesion progression. A healthy, *Lactobacillus*-dominant vaginal microbiota helps maintain immune stability, whereas a dysbiotic microbiota characterized by high diversity and anaerobic bacteria, e.g., CST-IV type, is associated with significant genital tract inflammation (Anahtar et al., 2015; Gholiof et al., 2022). This inflammatory environment is a strong predictor of persistent HPV infection and the progression of cervical lesions (Gholiof et al., 2022). The persistent presence of pro-inflammatory cytokines, e.g., IL-1β, TNF-α, may disrupt the tight junctions of the cervical epithelium and recruit large numbers of HIV-susceptible target cells, e.g., CCR5+ CD4+ T cells (Anahtar et al., 2015). Although this research primarily focused on HIV, the mechanism of inflammatory recruitment of target cells it revealed also helps us understand how disruption of the local immune environment impedes HPV clearance. The cervical microbiome in cervical cancer patients undergoes specific changes, such as a decrease in *Lactobacillus* abundance and an increase in the proportion of anaerobic bacteria (Gholiof et al., 2022). For example, one study found higher abundance of *Fusobacterium* spp. in cervical cancer samples, accompanied by elevated levels of immunosuppressive cytokines, e.g., IL-4, TGF-β1 (Audirac-Chalifour et al., 2016). These alterations in microbiota and immune profiles collectively create a microenvironment conducive to persistent HPV infection and the progression of cervical intraepithelial neoplasia (CIN) (Chambers et al., 2021). The association between dysbiosis and inflammation is not limited to HPV related cervical cancer, it is also prevalent in other gynecological malignancies. Taking endometrial cancer as an example, compared with women who are healthy or have benign lesions, patients with endometrial cancer exhibit reduced abundance of *Lactobacillus* in their uterine and vaginal microbiota, while the abundance of *Prevotella*, *Atopobium*, *Porphyromonas*, *Anaerococcus*, *Dialister*, and *Peptoniphilus* (Aquino et al., 2024; Walsh et al., 2019; Walther-António et al., 2016; Wang et al., 2022). Notably, the coexistence of *Atopobium* and *Porphyromonas*, along with a higher vaginal pH (>4.5), has been found to be closely associated with an increased risk of endometrial cancer (Aquino et al., 2024; Walther-António et al., 2016). Such abnormalities in microbial composition are not merely concomitant phenomena following tumor onset, the chronic inflammation they induce is considered a core driver of endometrial cancer development (Ganz, 2003). This persistent inflammatory state contributes to tumorigenesis and progression by generating free radicals, causing DNA damage, promoting abnormal cell proliferation, and inducing angiogenesis (Aquino et al., 2024; Ganz, 2003). Further clinical studies have revealed that the structural characteristics of the vaginal microbiota are even associated with tumor malignancy (Hakimjavadi et al., 2022). Microbiota α-diversity is positively correlated with the pathological grade of endometrial cancer, as lesions progress from benign uterine lesions to low-grade and high-grade endometrial cancer, the species richness and evenness of distribution in patients’ vaginal microbiota also increase (Aquino et al., 2024; Hakimjavadi et al., 2022). Different types of vaginal microbiome community states are also associated with tumor histological types and grades. This suggests that microbiome analysis not only aids in distinguishing between benign and malignant diseases but may also serve as a potential biomarker for predicting tumor aggressiveness (Aquino et al., 2024; Hakimjavadi et al., 2022). Collectively, these findings indicate that microbiome dysbiosis in the lower genital tract and uterus plays a significant role in the development and progression of gynecological cancers by shaping a persistent pro-inflammatory, pro-oncogenic microenvironment.

### 4.3 Endometriosis

Endometriosis, as a chronic inflammatory disease arises in part from the epithelial barrier impairment, immune pathway activation, and pro-inflammatory metabolite shifts induced by reproductive tract dysbiosis. Current evidence suggests a bidirectional association between this disease and dysbiosis of the genital and gut microbiota (Leonardi et al., 2020). Compared to healthy controls, the composition of the genital microbiota in patients with endometriosis has undergone significant changes (Jiang et al., 2021). Specifically, in the cervical and vaginal microbiota, the abundance of *Gardnerella*, *Shigella*, *Streptococcus*, *Escherichia coli*, and *Ureaplasma* increased, while the abundance of *Atopobacter* decreased (Ata et al., 2019). In the endometrial microbiota, the Actinobacteria phylum, Oxalobacteraceae and Streptococcaceae families were enriched (Wessels et al., 2021); whereas in ectopic lesions, there was a decrease in *Lactobacillus* and an increase in *Aliishwanella*, *Enterococcus*, and *Pseudomonas* (Hernandes et al., 2020). Therefore, microbiota dysbiosis is considered a key driver of endometriosis-associated inflammation. An animal study demonstrated that treatment with broad-spectrum antibiotics or metronidazole significantly reduced endometriotic lesions in mice and decreased pro-inflammatory cytokines IL-1β, IL-6, TNF-α, TGF-β1 in peritoneal fluid (Chadchan et al., 2019; Gholiof et al., 2022). This directly demonstrates that the microbiota is involved in the inflammatory response and growth processes of the lesions. In humans, specific bacterial species may drive lesion angiogenesis, proliferation, and immune evasion by activating immune cells, such as macrophages, and releasing pro-inflammatory factors, such as IL-1β and TNF-α (Gholiof et al., 2022).

### 4.4 Infertility and assisted reproductive outcomes

The three core inflammatory mechanisms, including epithelial barrier injury, immune disorder, metabolite imbalance, underlie the adverse effects of microbial dysbiosis on endometrial receptivity, embryo implantation, and assisted reproductive outcomes. The composition of the endometrial and vaginal microbiota has a significant impact on embryo implantation and the success rates of assisted reproductive technologies (ART) (Li et al., 2020), as the endometrium is not sterile. A key study demonstrated that, in women undergoing *in vitro* fertilization (IVF) with an endometrial microbiome dominated by non-lactobacilli have substantially lower rates of implantation, pregnancy, ongoing pregnancy and live birth (Moreno et al., 2016); conversely, an endometrial environment dominated by lactobacilli is more conducive to embryo implantation (Kyono et al., 2018; Moreno et al., 2016). Chronic endometritis is a benign, infection-related condition that frequently leads to poor outcomes in assisted reproduction (Park et al., 2016). When researchers transplanted the vaginal microbiota of patients with chronic endometritis into rats, it triggered severe uterine inflammation in the rats, suggesting that specific pathogenic microbial communities may be associated with recurrent implantation failure (Gholiof et al., 2022). Because the vagina is adjacent to the uterus, the state of its microbiota is predictive of the development of endometritis. When vaginal microbiota diversity is high and lactobacilli are absent, e.g., CST-IV type, this is often accompanied by genital tract inflammation, and such an environment may be unfavorable for maintaining pregnancy (Anahtar et al., 2015; Li et al., 2020). Additionally, in women with primary ovarian insufficiency (POF), vaginal microbiota diversity is typically higher, and the abundance of *Gardnerella*, *Prevotella*, and *Bacteroides* genera increases (Wang et al., 2020).

### 4.5 Adverse pregnancy outcomes

Microbial dysbiosis triggers adverse pregnancy outcomes mainly through the epithelial barrier disruption, immune inflammatory cascade, and metabolic disturbance pathways. Vaginal microbiota dysbiosis, particularly BV, is an independent risk factor for preterm birth and is associated with adverse pregnancy outcomes, like preterm birth (Li et al., 2020). During pregnancy, a healthy vaginal microbiota typically stabilizes and is dominated by lactobacilli (DiGiulio et al., 2015; Romero et al., 2014). However, an imbalanced vaginal microbiome during pregnancy, specifically an elevated level of bacterial diversity and the existence of anaerobic bacteria, could lead to cervical vaginitis, and this inflammatory state is a key step in triggering the preterm birth pathway (Short et al., 2021). It activates pattern recognition receptors on the surface of epithelial cells and immune cells through dysregulated microbiota and their metabolic by-products, thereby leading to elevated levels of pro-inflammatory cytokines, such as IL-1β, IL-6 and IL-8, and chemokines (Campisciano et al., 2018; Łaniewski et al., 2020; Villa et al., 2020). In HIV-infected pregnant women, vaginal microbiota diversity is higher and the inflammatory response is more pronounced, which is consistent with their increased risk of preterm birth (Short et al., 2021).

## 5 Conclusion

The FRT microbiota, dominated by *Lactobacillus*, is crucial for mucosal defense, immune balance and reproductive health. Disruption to the microbiota could compromise epithelial integrity, activate pro-inflammatory signals and alter metabolic profiles, creating a persistent inflammatory microenvironment that leads to gynecological disorders. The microbiota-immune-inflammation axis provides a common pathological basis for diseases such as BV, PID, persistent HPV infection, cervical malignancies, endometriosis, infertility and preterm birth. Furthermore, physiological, behavioral, and environmental factors dynamically regulate the FRT microbiota, underscoring the necessity of developing personalized health strategies. Modulating the FRT microbiome in targeted ways, such as restoring *Lactobacillus* dominance, correcting dysbiosis and alleviating chronic inflammation, represents a highly promising approach to improving the diagnosis, prevention and treatment of gynecological diseases. Future research should optimize microbiome-based biomarkers, develop precision interventions, and elucidate their long-term effects on systemic health to advance clinical translation in the field of reproductive medicine. Additionally, in recent years, artificial intelligence and multi-omics technologies have paved the way for the clinical application of the “microbiota-immune-inflammation” axis (Chen et al., 2025). Machine learning could analyze data on the reproductive tract microbiome and inflammation in order to develop models for precisely diagnosing and predicting the progression of gynecological diseases (Roy and Verma, 2025). Interpretable AI could identify the key mechanisms through which the microbiome regulates inflammation, thereby advancing personalized diagnosis and treatment (Yuan et al., 2024). Meanwhile, integrating multi-omics data helps to systematically elucidate host-microbe interaction mechanisms, accelerating the implementation of precision interventions based on microbiome modulation (Lin et al., 2025). Future efforts should focus on advancing the clinical validation of AI models to support the precise prevention and treatment of women’s reproductive health conditions.

## References

[B1] Alakomi H. L. Skyttä E. Saarela M. Mattila-Sandholm T. Latva-Kala K. Helander I. M. (2000). Lactic acid permeabilizes gram-negative bacteria by disrupting the outer membrane. *Appl. Environ. Microbiol.* 66 2001–2005. 10.1128/AEM.66.5.2001-2005.2000 10788373 PMC101446

[B2] Aldunate M. Srbinovski D. Hearps A. C. Latham C. F. Ramsland P. A. Gugasyan R. et al. (2015). Antimicrobial and immune modulatory effects of lactic acid and short chain fatty acids produced by vaginal microbiota associated with eubiosis and bacterial vaginosis. *Front. Physiol.* 6:164. 10.3389/fphys.2015.00164 26082720 PMC4451362

[B3] Alvarez-Olmos M. I. Barousse M. M. Rajan L. Van Der Pol B. J. Fortenberry D. Orr D. et al. (2004). Vaginal lactobacilli in adolescents: Presence and relationship to local and systemic immunity, and to bacterial vaginosis. *Sex. Transm. Dis.* 31 393–400. 10.1097/01.olq.0000130454.83883.e9 15215693

[B4] Amabebe E. Anumba D. O. C. (2020). Female gut and genital tract microbiota-induced crosstalk and differential effects of short-chain fatty acids on immune sequelae. *Front. Immunol.* 11:2184. 10.3389/fimmu.2020.02184 33013918 PMC7511578

[B5] Anahtar M. N. Byrne E. H. Doherty K. E. Bowman B. A. Yamamoto H. S. Soumillon M. et al. (2015). Cervicovaginal bacteria are a major modulator of host inflammatory responses in the female genital tract. *Immunity* 42 965–976. 10.1016/j.immuni.2015.04.019 25992865 PMC4461369

[B6] Aquino C. I. Nicosia A. Ligori A. Volpicelli A. I. Surico D. (2024). Microbiota status and endometrial cancer: A narrative review about possible correlations in affected versus healthy patients. *Sci* 6:75. 10.3390/sci6040075

[B7] Aroutcheva A. Gariti D. Simon M. Shott S. Faro J. Simoes J. A. et al. (2001). Defense factors of vaginal lactobacilli. *Am. J. Obstet. Gynecol.* 185 375–379. 10.1067/mob.2001.115867 11518895

[B8] Ata B. Yildiz S. Turkgeldi E. Brocal V. P. Dinleyici E. C. Moya A. et al. (2019). The endobiota study: Comparison of vaginal, cervical and gut microbiota between women with stage 3/4 endometriosis and healthy controls. *Sci. Rep.* 9:2204. 10.1038/s41598-019-39700-6 30778155 PMC6379373

[B9] Audirac-Chalifour A. Torres-Poveda K. Bahena-Román M. Téllez-Sosa J. Martínez-Barnetche J. Cortina-Ceballos B. et al. (2016). Cervical microbiome and cytokine profile at various stages of cervical cancer: A pilot study. *PLoS One* 11:e0153274. 10.1371/journal.pone.0153274 27115350 PMC4846060

[B10] Baker J. M. Chase D. M. Herbst-Kralovetz M. M. (2018). Uterine microbiota: Residents, tourists, or invaders? *Front. Immunol.* 9:208. 10.3389/fimmu.2018.00208 29552006 PMC5840171

[B11] Bradford L. L. Ravel J. (2017). The vaginal mycobiome: A contemporary perspective on fungi in women’s health and diseases. *Virulence* 8 342–351. 10.1080/21505594.2016.1237332 27657355 PMC5411243

[B12] Bradshaw C. S. Morton A. N. Hocking J. Garland S. M. Morris M. B. Moss L. M. et al. (2006). High recurrence rates of bacterial vaginosis over the course of 12 months after oral metronidazole therapy and factors associated with recurrence. *J. Infect. Dis.* 193 1478–1486. 10.1086/503780 16652274

[B13] Bradshaw C. S. Sobel J. D. (2016). Current treatment of bacterial vaginosis-limitations and need for innovation. *J. Infect. Dis.* 214 (Suppl. 1), S14–S20. 10.1093/infdis/jiw159 27449869 PMC4957510

[B14] Brotman R. M. Shardell M. D. Gajer P. Fadrosh D. Chang K. Silver M. I. et al. (2018). Association between the vaginal microbiota, menopause status, and signs of vulvovaginal atrophy. *Menopause* 25 1321–1330. 10.1097/GME.0000000000001236 30358729

[B15] Campisciano G. Zanotta N. Licastro D. De Seta F. Comar M. (2018). In vivo microbiome and associated immune markers: New insights into the pathogenesis of vaginal dysbiosis. *Sci. Rep.* 8:2307. 10.1038/s41598-018-20649-x 29396486 PMC5797242

[B16] Carvalho F. A. Aitken J. D. Vijay-Kumar M. Gewirtz A. T. (2012). Toll-like receptor-gut microbiota interactions: Perturb at your own risk! *Annu. Rev. Physiol.* 74 177–198. 10.1146/annurev-physiol-020911-153330 22035346

[B17] Cerca N. Martins S. Cerca F. Jefferson K. K. Pier G. B. Oliveira R. et al. (2005). Comparative assessment of antibiotic susceptibility of coagulase-negative staphylococci in biofilm versus planktonic culture as assessed by bacterial enumeration or rapid XTT colorimetry. *J. Antimicrob. Chemother.* 56 331–336. 10.1093/jac/dki217 15980094 PMC1317301

[B18] Chadchan S. B. Cheng M. Parnell L. A. Yin Y. Schriefer A. Mysorekar I. U. et al. (2019). Antibiotic therapy with metronidazole reduces endometriosis disease progression in mice: A potential role for gut microbiota. *Hum. Reprod.* 34 1106–1116. 10.1093/humrep/dez041 31037294 PMC6554192

[B19] Chambers L. M. Bussies P. Vargas R. Esakov E. Tewari S. Reizes O. et al. (2021). The microbiome and gynecologic cancer: Current evidence and future opportunities. *Curr. Oncol. Rep.* 23:92. 10.1007/s11912-021-01079-x 34125319

[B20] Chee W. J. Y. Chew S. Y. Than L. T. L. (2020). Vaginal microbiota and the potential of *Lactobacillus* derivatives in maintaining vaginal health. *Microb. Cell Fact.* 19:203. 10.1186/s12934-020-01464-4 33160356 PMC7648308

[B21] Chen A.-T. Zhang Y. Zhang J. (2025). Artificial intelligence in cardiac metabolism: The next frontier in cardiovascular health. *Metab. Target Organ Damage* 5:3. 10.20517/mtod.2024.82

[B22] Chen C. Song X. Wei W. Zhong H. Dai J. Lan Z. et al. (2017). The microbiota continuum along the female reproductive tract and its relation to uterine-related diseases. *Nat. Commun.* 8:875. 10.1038/s41467-017-00901-0 29042534 PMC5645390

[B23] Dabee S. Passmore J. S. Heffron R. Jaspan H. B. (2021). The complex link between the female genital microbiota, genital infections, and inflammation. *Infect. Immun.* 89:e00487-20. 10.1128/IAI.00487-20 33558324 PMC8091093

[B24] Delgado-Diaz D. J. Tyssen D. Hayward J. A. Gugasyan R. Hearps A. C. Tachedjian G. (2020). Distinct immune responses elicited from cervicovaginal epithelial cells by lactic acid and short chain fatty acids associated with optimal and non-optimal vaginal microbiota. *Front. Cell. Infect. Microbiol.* 9:446. 10.3389/fcimb.2019.00446 31998660 PMC6965070

[B25] DiGiulio D. B. Callahan B. J. McMurdie P. J. Costello E. K. Lyell D. J. Robaczewska A. et al. (2015). Temporal and spatial variation of the human microbiota during pregnancy. *Proc. Natl. Acad. Sci. U. S. A.* 112 11060–11065. 10.1073/pnas.1502875112 26283357 PMC4568272

[B26] Eschenbach D. A. Thwin S. S. Patton D. L. Hooton T. M. Stapleton A. E. Agnew K. et al. (2000). Influence of the normal menstrual cycle on vaginal tissue, discharge, and microflora. *Clin. Infect. Dis.* 30 901–907. 10.1086/313818 10852812

[B27] Fazeli A. Bruce C. Anumba D. O. (2005). Characterization of toll-like receptors in the female reproductive tract in humans. *Hum. Reprod.* 20 1372–1378. 10.1093/humrep/deh775 15695310

[B28] France M. T. Ma B. Gajer P. Brown S. Humphrys M. S. Holm J. B. et al. (2020). VALENCIA: A nearest centroid classification method for vaginal microbial communities based on composition. *Microbiome* 8:166. 10.1186/s40168-020-00934-6 33228810 PMC7684964

[B29] Furusawa Y. Obata Y. Fukuda S. Endo T. A. Nakato G. Takahashi D. et al. (2013). Commensal microbe-derived butyrate induces the differentiation of colonic regulatory T cells. *Nature* 504 446–450. 10.1038/nature12721 24226770

[B30] Gajer P. Brotman R. M. Bai G. Sakamoto J. Schütte U. M. Zhong X. et al. (2012). Temporal dynamics of the human vaginal microbiota. *Sci. Transl. Med.* 4:132ra52. 10.1126/scitranslmed.3003605 22553250 PMC3722878

[B31] Ganz T. (2003). Defensins: Antimicrobial peptides of innate immunity. *Nat. Rev. Immunol.* 3 710–720. 10.1038/nri1180 12949495

[B32] Genc M. R. Vardhana S. Delaney M. L. Onderdonk A. Tuomala R. Norwitz E. et al. (2004). Relationship between a toll-like receptor-4 gene polymorphism, bacterial vaginosis-related flora and vaginal cytokine responses in pregnant women. *Eur. J. Obstet. Gynecol. Reprod. Biol.* 116 152–156. 10.1016/j.ejogrb.2004.02.010 15358455

[B33] Gholiof M. Adamson-De Luca E. Wessels J. M. (2022). The female reproductive tract microbiotas, inflammation, and gynecological conditions. *Front. Reprod. Health* 4:963752. 10.3389/frph.2022.963752 36303679 PMC9580710

[B34] Goldenberg R. L. Culhane J. F. Iams J. D. Romero R. (2008). Epidemiology and causes of preterm birth. *Lancet* 371 75–84. 10.1016/S0140-6736(08)60074-4 18177778 PMC7134569

[B35] Greenbaum S. Greenbaum G. Moran-Gilad J. Weintraub A. Y. (2019). Ecological dynamics of the vaginal microbiome in relation to health and disease. *Am. J. Obstet. Gynecol.* 220 324–335. 10.1016/j.ajog.2018.11.1089 30447213

[B36] Hakimjavadi H. George S. H. Taub M. Dodds L. V. Sanchez-Covarrubias A. P. Huang M. et al. (2022). The vaginal microbiome is associated with endometrial cancer grade and histology. *Cancer Res. Commun.* 2 447–455. 10.1158/2767-9764.CRC-22-0075 35928983 PMC9345414

[B37] Hansen L. K. Becher N. Bastholm S. Glavind J. Ramsing M. Kim C. J. et al. (2014). The cervical mucus plug inhibits, but does not block, the passage of ascending bacteria from the vagina during pregnancy. *Acta Obstet. Gynecol. Scand.* 93 102–108. 10.1111/aogs.12296 24266587 PMC5987199

[B38] Happel A. U. Varsani A. Balle C. Passmore J. A. Jaspan H. (2020). The vaginal virome-balancing female genital tract bacteriome, mucosal immunity, and sexual and reproductive health outcomes? *Viruses* 12:832. 10.3390/v12080832 32751611 PMC7472209

[B39] Hebel K. Rudolph M. Kosak B. Chang H. D. Butzmann J. Brunner-Weinzierl M. C. (2011). IL-1β and TGF-β act antagonistically in induction and differentially in propagation of human proinflammatory precursor CD4+ T cells. *J. Immunol.* 187 5627–5635. 10.4049/jimmunol.1003998 22048775

[B40] Hernandes C. Silveira P. Rodrigues Sereia A. F. Christoff A. P. Mendes H. Valter de Oliveira L. F. et al. (2020). Microbiome profile of deep endometriosis patients: Comparison of vaginal fluid, endometrium and lesion. *Diagnostics* 10:163. 10.3390/diagnostics10030163 32192080 PMC7151170

[B41] Horne A. W. Stock S. J. King A. E. (2008). Innate immunity and disorders of the female reproductive tract. *Reproduction* 135 739–749. 10.1530/REP-07-0564 18502890

[B42] Jiang I. Yong P. J. Allaire C. Bedaiwy M. A. (2021). Intricate connections between the microbiota and endometriosis. *Int. J. Mol. Sci.* 22:5644. 10.3390/ijms22115644 34073257 PMC8198999

[B43] Kenyon C. Colebunders R. Crucitti T. (2013). The global epidemiology of bacterial vaginosis: A systematic review. *Am. J. Obstet. Gynecol.* 209 505–523. 10.1016/j.ajog.2013.05.006 23659989

[B44] Klynstra F. B. van der Laan E. J. Linders H. J. (1967). Methods for the separation of acid mucopolysaccharides of the human aorta. *J. Atheroscler. Res.* 7 257–264. 10.1016/s0368-1319(67)80087-5 4226773

[B45] Kyono K. Hashimoto T. Nagai Y. Sakuraba Y. (2018). Analysis of endometrial microbiota by 16S ribosomal RNA gene sequencing among infertile patients: A single-center pilot study. *Reprod. Med. Biol.* 17 297–306. 10.1002/rmb2.12105 30013432 PMC6046523

[B46] Łaniewski P. Ilhan Z. E. Herbst-Kralovetz M. M. (2020). The microbiome and gynaecological cancer development, prevention and therapy. *Nat. Rev. Urol.* 17 232–250. 10.1038/s41585-020-0286-z 32071434 PMC9977514

[B47] Lennard K. Dabee S. Barnabas S. L. Havyarimana E. Blakney A. Jaumdally S. Z. et al. (2017). Microbial composition predicts genital tract inflammation and persistent bacterial vaginosis in South African adolescent females. *Infect. Immun.* 86:e00410-17. 10.1128/IAI.00410-17 29038128 PMC5736802

[B48] Leonardi M. Hicks C. El-Assaad F. El-Omar E. Condous G. (2020). Endometriosis and the microbiome: A systematic review. *BJOG* 127 239–249. 10.1111/1471-0528.15916 31454452

[B49] Li H. Zang Y. Wang C. Li H. Fan A. Han C. et al. (2020). The interaction between microorganisms, metabolites, and immune system in the female genital tract microenvironment. *Front. Cell. Infect. Microbiol.* 10:609488. 10.3389/fcimb.2020.609488 33425785 PMC7785791

[B50] Lin Z. Qiang J. Gu Y. Li H. (2025). Artificial intelligence in ovarian cancer: Current advances and perspectives. *Med. Adv.* 3 256–267. 10.1002/med4.70036

[B51] Madere F. S. Monaco C. L. (2022). The female reproductive tract virome: Understanding the dynamic role of viruses in gynecological health and disease. *Curr. Opin. Virol.* 52 15–23. 10.1016/j.coviro.2021.10.010 34800892 PMC8844092

[B52] Marrazzo J. M. Koutsky L. A. Eschenbach D. A. Agnew K. Stine K. Hillier S. L. (2002). Characterization of vaginal flora and bacterial vaginosis in women who have sex with women. *J. Infect. Dis.* 185 1307–1313. 10.1086/339884 12001048

[B53] Mirmonsef P. Zariffard M. R. Gilbert D. Makinde H. Landay A. L. Spear G. T. (2012). Short-chain fatty acids induce pro-inflammatory cytokine production alone and in combination with toll-like receptor ligands. *Am. J. Reprod. Immunol.* 67 391–400. 10.1111/j.1600-0897.2011.01089.x 22059850 PMC3288536

[B54] Mitchell C. Marrazzo J. (2014). Bacterial vaginosis and the cervicovaginal immune response. *Am. J. Reprod. Immunol.* 71 555–563. 10.1111/aji.12264 24832618 PMC4128638

[B55] Molina N. M. Sola-Leyva A. Saez-Lara M. J. Plaza-Diaz J. Tubić-Pavlović A. Romero B. et al. (2020). New opportunities for endometrial health by modifying uterine microbial composition: Present or future? *Biomolecules* 10:593. 10.3390/biom10040593 32290428 PMC7226034

[B56] Moosa Y. Kwon D. de Oliveira T. Wong E. B. (2020). Determinants of vaginal microbiota composition. *Front. Cell. Infect. Microbiol.* 10:467. 10.3389/fcimb.2020.00467 32984081 PMC7492712

[B57] Moreno I. Codoñer F. M. Vilella F. Valbuena D. Martinez-Blanch J. F. Jimenez-Almazán J. et al. (2016). Evidence that the endometrial microbiota has an effect on implantation success or failure. *Am. J. Obstet. Gynecol.* 215 684–703. 10.1016/j.ajog.2016.09.075 27717732

[B58] O’Hanlon D. E. Gajer P. Brotman R. M. Ravel J. (2020). Asymptomatic bacterial vaginosis is associated with depletion of mature superficial cells shed from the vaginal epithelium. *Front. Cell. Infect. Microbiol.* 10:106. 10.3389/fcimb.2020.00106 32211347 PMC7076050

[B59] O’Hanlon D. E. Moench T. R. Cone R. A. (2011). In vaginal fluid, bacteria associated with bacterial vaginosis can be suppressed with lactic acid but not hydrogen peroxide. *BMC Infect. Dis.* 11:200. 10.1186/1471-2334-11-200 21771337 PMC3161885

[B60] O’Hanlon D. E. Moench T. R. Cone R. A. (2013). Vaginal pH and microbicidal lactic acid when lactobacilli dominate the microbiota. *PLoS One* 8:e80074. 10.1371/journal.pone.0080074 24223212 PMC3819307

[B61] Park H. J. Kim Y. S. Yoon T. K. Lee W. S. (2016). Chronic endometritis and infertility. *Clin. Exp. Reprod. Med.* 43 185–192. 10.5653/cerm.2016.43.4.185 28090456 PMC5234283

[B62] Petrova M. I. Reid G. Vaneechoutte M. Lebeer S. (2017). *Lactobacillus iners*: Friend or foe? *Trends Microbiol.* 25 182–191. 10.1016/j.tim.2016.11.007 27914761

[B63] Radtke A. L. Quayle A. J. Herbst-Kralovetz M. M. (2012). Microbial products alter the expression of membrane-associated mucin and antimicrobial peptides in a three-dimensional human endocervical epithelial cell model. *Biol. Reprod.* 87:132. 10.1095/biolreprod.112.103366 23053434 PMC4435425

[B64] Ravel J. Brotman R. M. Gajer P. Ma B. Nandy M. Fadrosh D. W. et al. (2013). Daily temporal dynamics of vaginal microbiota before, during and after episodes of bacterial vaginosis. *Microbiome* 1:29. 10.1186/2049-2618-1-29 24451163 PMC3968321

[B65] Ravel J. Gajer P. Abdo Z. Schneider G. M. Koenig S. S. McCulle S. L. et al. (2011). Vaginal microbiome of reproductive-age women. *Proc. Natl. Acad. Sci. U. S. A.* 108 (Suppl. 1), 4680–4687. 10.1073/pnas.1002611107 20534435 PMC3063603

[B66] Romero R. Hassan S. S. Gajer P. Tarca A. L. Fadrosh D. W. Nikita L. et al. (2014). The composition and stability of the vaginal microbiota of normal pregnant women is different from that of non-pregnant women. *Microbiome* 2:4. 10.1186/2049-2618-2-4 24484853 PMC3916806

[B67] Roselletti E. Sabbatini S. Perito S. Mencacci A. Vecchiarelli A. Monari C. (2020). Apoptosis of vaginal epithelial cells in clinical samples from women with diagnosed bacterial vaginosis. *Sci. Rep.* 10:1978. 10.1038/s41598-020-58862-2 32029862 PMC7005030

[B68] Roy A. Verma N. (2025). Machine learning models and AI in predicting diagnosis and prognosis in alcohol-related and metabolic dysfunction-associated steatotic liver disease. *Metab. Target Organ Damage* 5:8. 10.20517/mtod.2024.84

[B69] Santiago G. L. Cools P. Verstraelen H. Trog M. Missine G. El Aila N. et al. (2011). Longitudinal study of the dynamics of vaginal microflora during two consecutive menstrual cycles. *PLoS One* 6:e28180. 10.1371/journal.pone.0028180 22140538 PMC3227645

[B70] Shen J. Song N. Williams C. J. Brown C. J. Yan Z. Xu C. et al. (2016). Erratum: Effects of low dose estrogen therapy on the vaginal microbiomes of women with atrophic vaginitis. *Sci. Rep.* 6:34119. 10.1038/srep34119 27897166 PMC5126675

[B71] Short C. S. Brown R. G. Quinlan R. Lee Y. S. Smith A. Marchesi J. R. et al. (2021). *Lactobacillus*-depleted vaginal microbiota in pregnant women living with HIV-1 infection are associated with increased local inflammation and preterm birth. *Front. Cell. Infect. Microbiol.* 10:596917. 10.3389/fcimb.2020.596917 33643930 PMC7905210

[B72] Srinivasan S. Liu C. Mitchell C. M. Fiedler T. L. Thomas K. K. Agnew K. J. et al. (2010). Temporal variability of human vaginal bacteria and relationship with bacterial vaginosis. *PLoS One* 5:e10197. 10.1371/journal.pone.0010197 20419168 PMC2855365

[B73] Turpin R. Slopen N. Borgogna J. C. Yeoman C. J. He X. Miller R. S. et al. (2021). Perceived stress and molecular bacterial vaginosis in the national institutes of health longitudinal study of vaginal flora. *Am. J. Epidemiol.* 190 2374–2383. 10.1093/aje/kwab147 34008013 PMC8799897

[B74] Usluogullari B. Gumus I. Gunduz E. Kaygusuz I. Simavli S. Acar M. et al. (2014). The role of human dectin-1 Y238X gene polymorphism in recurrent vulvovaginal candidiasis infections. *Mol. Biol. Rep.* 41 6763–6768. 10.1007/s11033-014-3562-2 25008994

[B75] van de Wijgert J. H. H. M. Jespers V. (2017). The global health impact of vaginal dysbiosis. *Res. Microbiol.* 168 859–864. 10.1016/j.resmic.2017.02.003 28257809

[B76] Verwijs M. C. Agaba S. K. Darby A. C. van de Wijgert J. H. H. M. (2020). Impact of oral metronidazole treatment on the vaginal microbiota and correlates of treatment failure. *Am. J. Obstet. Gynecol.* 222 157.e1–157.e13. 10.1016/j.ajog.2019.08.008 31404542 PMC6995998

[B77] Villa P. Cipolla C. D’Ippolito S. Amar I. D. Shachor M. Ingravalle F. et al. (2020). The interplay between immune system and microbiota in gynecological diseases: A narrative review. *Eur. Rev. Med. Pharmacol. Sci.* 24 5676–5690. 10.26355/eurrev_202005_21359 32495903

[B78] Walsh D. M. Hokenstad A. N. Chen J. Sung J. Jenkins G. D. Chia N. et al. (2019). Postmenopause as a key factor in the composition of the Endometrial Cancer Microbiome (ECbiome). *Sci. Rep.* 9:19213. 10.1038/s41598-019-55720-8 31844128 PMC6915778

[B79] Walther-António M. R. Chen J. Multinu F. Hokenstad A. Distad T. J. Cheek E. H. et al. (2016). Potential contribution of the uterine microbiome in the development of endometrial cancer. *Genome Med.* 8:122. 10.1186/s13073-016-0368-y 27884207 PMC5123330

[B80] Wang J. Li Z. Ma X. Du L. Jia Z. Cui X. et al. (2021). Translocation of vaginal microbiota is involved in impairment and protection of uterine health. *Nat. Commun.* 12:4191. 10.1038/s41467-021-24516-8 34234149 PMC8263591

[B81] Wang J. Xu J. Han Q. Chu W. Lu G. Chan W. Y. et al. (2020). Changes in the vaginal microbiota associated with primary ovarian failure. *BMC Microbiol.* 20:230. 10.1186/s12866-020-01918-0 32727366 PMC7392721

[B82] Wang L. Yang J. Su H. Shi L. Chen B. Zhang S. (2022). Endometrial microbiota from endometrial cancer and paired pericancer tissues in postmenopausal women: Differences and clinical relevance. *Menopause* 29 1168–1175. 10.1097/GME.0000000000002053 36150116 PMC9512232

[B83] Wessels J. M. Domínguez M. A. Leyland N. A. Agarwal S. K. Foster W. G. (2021). Endometrial microbiota is more diverse in people with endometriosis than symptomatic controls. *Sci. Rep.* 11:18877. 10.1038/s41598-021-98380-3 34556738 PMC8460742

[B84] Witkin S. S. Linhares I. M. (2017). Why do lactobacilli dominate the human vaginal microbiota? *BJOG* 124 606–611. 10.1111/1471-0528.14390 28224747

[B85] Witkin S. S. Linhares I. M. Giraldo P. (2007). Bacterial flora of the female genital tract: Function and immune regulation. *Best Pract. Res. Clin. Obstet. Gynaecol.* 21 347–354. 10.1016/j.bpobgyn.2006.12.004 17215167

[B86] Yuan H. Yu K. Xie F. Liu M. Sun S. (2024). Automated machine learning with interpretation: A systematic review of methodologies and applications in healthcare. *Med. Adv.* 2 205–237. 10.1002/med4.75

[B87] Zhou X. Brown C. J. Abdo Z. Davis C. C. Hansmann M. A. Joyce P. et al. (2007). Differences in the composition of vaginal microbial communities found in healthy Caucasian and black women. *ISME J.* 1 121–133. 10.1038/ismej.2007.12 18043622

